# Cultivar-Specific Differences in C6 and C7 Sugar Metabolism During Avocado Ripening: Comparative Insights from *Bacon*, *Fuerte*, and *Hass*

**DOI:** 10.3390/plants14182856

**Published:** 2025-09-12

**Authors:** María Gemma Beiro-Valenzuela, Romina P. Monasterio, Irene Serrano-García, Elena Hurtado-Fernández, Carmen María Sánchez-Arévalo, Jorge Fernando Fernández-Sánchez, Romina Pedreschi, Lucía Olmo-García, Alegría Carrasco-Pancorbo

**Affiliations:** 1 Department of Analytical Chemistry, Faculty of Sciences, University of Granada, Ave. Fuentenueva s/n, 18071 Granada, Spain; gemabv@ugr.es (M.G.B.-V.); rmonasterio@mendoza-conicet.gob.ar (R.P.M.); iserrano@ugr.es (I.S.-G.); mamens@ugr.es (C.M.S.-A.); jffernan@ugr.es (J.F.F.-S.); luciaolmo@ugr.es (L.O.-G.); 2 Instituto de Biología Agrícola de Mendoza (IBAM), UNCuyo—CONICET, Facultad de Ciencias Agrarias, Chacras de Coria, Mendoza 5505, Argentina; 3 Department of Biological and Health Sciences, Faculty of Health Sciences, Loyola University (Universidad Loyola Andalucía), Avda. de las Universidades s/n, 41704 Dos Hermanas, Spain; emhurtado@uloyola.es; 4 Escuela de Agronomía, Facultad de Ciencias Agronómicas y de los Alimentos, Pontificia Universidad Católica de Valparaíso, Calle San Francisco S/N, La Palma, Quillota 2260000, Chile; romina.pedreschi@pucv.cl; 5 Millennium Institute Center for Genome Regulation (CRG), Santiago 8331150, Chile

**Keywords:** avocado ripening, non-structural carbohydrates, hydrophilic interaction chromatography–mass spectrometry, *Persea americana*

## Abstract

Avocado is a unique fruit in which of seven-carbon (C7) sugars (notably *D*-mannoheptulose and perseitol) dominate the carbohydrate profile at harvest. Despite growing interest in sugar-mediated ripening processes, limited comparative data exist across cultivars. This work characterises the dynamic changes in non-structural carbohydrates in the mesotecarp of three commercially relevant avocado varieties—*Bacon*, *Fuerte*, and *Hass*—across four defined ripening stages, from unripe to overripe, with five biological replicates per stage. Using a validated hydrophilic interaction liquid chromatography–mass spectrometry (HILIC–MS) method, we quantified five key sugars and assessed their evolution through ripening. Concentrations varied among the studied samples within the following ranges: *D*-mannoheptulose, 0.4–49 mg/g dry weight (DW); perseitol, 0.5–23 mg/g DW; glucose, 0.8–5.3 mg/g DW; fructose, 0.6–4.5 mg/g DW; and sucrose, 0.5–3.4 mg/g DW. C7 sugar levels consistently declined, while C6 sugars increased—primarily between the intermediate and ready-to-eat stages—with distinct cultivar-specific patterns. *Bacon* maintained elevated C7 concentrations for a longer period; *Fuerte* exhibited a rapid transition from C7 to C6 sugars; and *Hass* displayed a more gradual and balanced shift. Multivariate analysis (partial least squares discriminant analysis, PLS-DA) effectively discriminated between cultivars at each ripening stage, confirming cultivar-specific metabolic signatures. These findings offer new insights into avocado carbohydrate metabolism, emphasising variety-dependent pathways that could inform breeding strategies, optimise postharvest ripening protocols, and support the nutritional characterisation of different avocado cultivars.

## 1. Introduction

Avocado (*Persea americana* Mill.) is a subtropical evergreen fruit tree crop belonging to the Lauraceae family and originating in Mesoamerica, where it was consumed more than 10,000 years ago. Today, avocado is one of the most economically significant subtropical and tropical fruits worldwide, with global production exceeding ten million tons in 2023 and continuing to rise [1]. The market is dominated by the *Hass* cultivar, although other varieties such as *Bacon* and *Fuerte* remain commercially relevant in certain regions.

Being a climacteric fruit, the development of avocado fruit can be clearly divided into two distinct processes: fruit maturation and postharvest ripening. Maturation refers to the growth process that occurs while the fruit is still on the tree, and usually takes place between 20 and 60 weeks after pollination [2]. On the other hand, postharvest ripening involves the softening of the mesocarp and improvement of organoleptic properties [2,3], which occurs only after the fruit has been removed from the tree. Ripening, as a broader concept, involves a series of complex processes that result in changes in colour, flavour and texture, making the fruit palatable for consumption [4]. This transformation usually takes about seven days after harvesting at 25 °C [5] and involves numerous catabolic and anabolic modifications that require significant energy expenditure. Ultimately, the onset of tissue senescence leads to a condition of overripeness [4]. The duration of this process varies considerably among cultivars and is strongly influenced by the physiological maturity of the fruit at harvest. This variability reflects, in part, differences in biomass composition, particularly the proportions of cell wall polysaccharides, soluble carbohydrates, and other metabolites, which modulate resistance to biotic and abiotic stresses during growth, development, and postharvest ripening. Biomass composition also shapes sensory and nutritional attributes, including taste and potential health benefits. In most fleshy fruits, the primary components of dry biomass are cell wall constituents and non-structural carbohydrates [6], although in avocado the mesocarp is also notable for its exceptionally high lipid content. The importance of sugars in plants extends beyond their role as carbon skeletons and energy sources; they are also fundamental regulators of fruit development. Sugars influence texture, flavour, colour, and nutritional quality [7] and modulate numerous developmental processes, including embryogenesis, seed germination, stress responses, and both vegetative and reproductive growth [8,9]. Moreover, they protect proteins, lipids, and DNA from oxidative damage, thereby preventing cell death [10].

In avocado, soluble sugars represent over 40% of the mesocarp dry weight during early fruit growth and development [11], a distinctive trait largely attributable to the substantial presence of C7 sugars—*D*-mannoheptulose and its polyol form, perseitol. Unlike most fruits, which rely on C6 sugars such as glucose, fructose, and sucrose as their primary energy source during ripening, avocados accumulate and utilise C7 sugars as their principal carbohydrates during this phase [12,13,14].

Multiple physiological roles have been proposed for C7 sugars, although their precise contributions to energy metabolism and ripening regulation are not yet fully understood. Perseitol is widely considered a storage carbohydrate and energy reservoir, consistent with its high concentrations in seeds and declining levels in other tissues during maturation [15,16]. In contrast, *D*-mannoheptulose functions primarily as a transport sugar and likely supports energy-requiring biosynthetic processes in developing tissues [3,14,17,18,19]. Moreover, *D*-mannoheptulose is a potent inhibitor of hexokinase [13,19,20,21], a key glycolytic enzyme. This inhibition may reduce glucose phosphorylation, thereby delaying glycolytic flux and ripening progression [22]; however, this is likely one among several interacting regulatory mechanisms. In parallel, other sugars play more conventional roles: sucrose is thought to be the predominant transport form during preharvest stages [23]; glucose is essential for cellulose biosynthesis; and fructose contributes to lipogenesis, particularly during the oil accumulation phase [24]. Nonetheless, key questions remain regarding the respective roles of these sugars in postharvest energy metabolism, and how these metabolic patterns vary among avocado cultivars with distinct physiological traits and ripening behaviours.

The balance between C6 and C7 sugars reflects key metabolic shifts and differs significantly among cultivars. Recognising these varietal differences is essential, as avocados are often subjected to uniform postharvest handling, despite the fact that cultivar-specific sugar dynamics can influence ripening behaviour, shelf life, and overall postharvest quality. For instance, substantial differences have been reported among cultivars in fatty acid composition, as well as in mineral content, phytosterol levels, and tocopherol profiles—all of which underscore the unique metabolic characteristics of each variety [25]. A deeper understanding of these differences would not only support the development of more tailored postharvest strategies but also underscore the importance of investigating additional cultivars harvested at different times to better meet market demands.

Previous studies (summarised in Table S1—Supplementary) have examined the role of C6 and C7 sugars in avocado tissues and their potential contribution to ripening physiology. Valuable and rigorous prior research has provided important insights into sugar-mediated ripening control in avocado; however, the extent to which these dynamics differ across genotypes remains largely unexplored. Comparative analyses across multiple cultivars and clearly defined ripening stages are particularly scarce, hindering a comprehensive understanding of how these sugars modulate avocado physiology and quality attributes during softening. The cultivars *Bacon*, *Fuerte*, and *Hass* are widely cultivated and commercially important, yet they exhibit contrasting physiological and biochemical ripening behaviours. *Hass* is known for its extended postharvest life and well-characterised oil and sugar dynamics. In contrast, *Bacon* and *Fuerte* represent less-studied varieties with distinct mesocarp physiology and shelf-life characteristics. Studying these three cultivars provides a model system for comparing sugar-mediated regulation under genetically distinct backgrounds. Addressing this knowledge gap is crucial, as sugar dynamics not only underpin key metabolic shifts but also influence flavour, nutritional value, and postharvest behaviour—factors of increasing relevance to breeding, storage, and marketing strategies.

In this research, we hypothesise that differences in the C6 and C7 sugar profiles of different avocado cultivars modulate the metabolic trajectory of ripening, and that these profiles may serve as physiological markers of the stage of ripening or postharvest performance. Therefore, this study aims to systematically characterise the quantitative dynamics of non-structural carbohydrates (C6 and C7 sugars) in the mesocarp of three commercially important avocado cultivars—*Bacon*, *Fuerte*, and *Hass*—across four ripening stages. By applying a validated hydrophilic interaction liquid chromatography–mass spectrometry (HILIC–MS) approach under controlled conditions, we provide the first systematic comparison of cultivar-specific metabolic shifts throughout avocado ripening. If further studies continue along the lines explored in this work, these findings would help establish a valuable metabolic framework that could inform the optimisation of ripening protocols, guide the selection of cultivars with desirable sensory and nutritional attributes, and contribute to a deeper understanding of avocado fruit physiology.

## 2. Results and Discussion

Fruit growth and ripening are complex developmental processes influenced by environmental and physiological factors, yet their molecular regulation remains only partially elucidated. In most fleshy fruits, sucrose and other C6 sugars imported from leaves constitute the principal carbon and energy sources during ripening [23,26]. Avocado, by contrast, exhibits a distinctive metabolic profile in which C7 carbohydrates predominate in the mesocarp at harvest. Whereas the sensory and nutritional quality of many fruits depends largely on their sugar–acid balance, avocado quality is primarily determined by oil content and fatty acid composition. Nevertheless, monosaccharides play a fundamental role in primary metabolism, supplying energy, and biosynthetic precursors. This unusual carbohydrate composition makes avocado a valuable model for investigating sugar dynamics during ripening and highlights the need to understand how these profiles evolve across developmental stages and differ among cultivars.

### 2.1. Quantitative Profiles and Fluctuation Trends of Individual Sugars During Ripening

The five essential non-structural carbohydrates (fructose, glucose, *D*-mannoheptulose, perseitol, and sucrose) were quantified across 60 avocado samples (comprising 5 biological replicates × 4 ripening stages × 3 cultivars). Each sample was analysed in duplicate (preparing 2 technical replicates), resulting in a total of 120 injected extracts. Figure S1—Supplementary shows the extracted ion chromatograms (EICs) of the non-structural carbohydrates obtained from (A) a standard mixture and (B) a representative avocado mesocarp extract. Table 1 presents the elution order of compounds, retention time, detected *m*/*z*, molecular formula, calibration curve, coefficient of determination (r^2^), and linear range for each analyte. Calibration curves exhibited excellent linearity (r^2^ > 0.99) across the evaluated ranges. For *D*-mannoheptulose, two linear ranges were applied to account for the wide variability in its concentrations among avocado samples.

The analyte concentrations found in the mesocarp of *Hass*, *Fuerte*, and *Bacon* avocados throughout the softening process are presented in Table 2 (for clarity, Figure S2—Supplementary is also provided, presenting heatmaps of sugar profiles for each avocado variety). As outlined in the Materials and Methods section, a complementary dual-platform strategy was adopted to maximise analytical accuracy and reproducibility. High-resolution QTOF-MS was employed for the unambiguous identification of all target sugars, using accurate mass measurements and MS/MS fragmentation patterns. The availability of pure analytical standards for each compound enabled confident structural confirmation and alignment of retention times across platforms. Once compound identity was unequivocally established and chromatographic retention times were verified as consistent between systems, quantification was performed using ion trap LC-MS under the same HILIC conditions. The ion trap system was selected for its robust performance in routine quantification and its ability to handle biological replicates with high reproducibility. As both platforms produced concordant chromatograms for standards and sample matrices, this two-step workflow ensured both high-confidence identification and reliable quantification.

At harvest, *Bacon* fruit exhibited the highest total concentration of C7 sugars among the three cultivars, with *D*-mannoheptulose accounting for approximately 49 mg/g DW and perseitol contributing around 10 mg/g DW. Across ripening stages, *D*-mannoheptulose concentrations decreased significantly (*p* < 0.05), reaching approximately 10 mg/g DW at the ready-to-eat stage. Perseitol also showed a statistically significant reduction over time, though the decline was less pronounced from unripe to intermediate ripening, with concentrations remaining near 2 and 0.5 mg/g DW in ready-to-eat and overripe stages, respectively. In contrast, initial concentrations of C6 sugars (glucose, fructose, sucrose) were low (<2.5 mg/g DW each) but increased progressively during ripening. Glucose and fructose peaked at approximately 5 mg/g DW (overripe stage), while sucrose reached ~3 mg/g DW by the ready-to-eat stage. Notably, *Bacon* maintained relatively balanced sugar levels at the overripe stage, with residual C7 sugars and moderate C6 accumulation, indicating a moderated shift in carbohydrate composition and suggesting a more gradual metabolic transition compared to the other cultivars.

*Fuerte* mesocarp exhibited a distinct profile in which perseitol was the most abundant C7 sugar at the unripe stage (~23 mg/g DW), followed by *D*-mannoheptulose (~20 mg/g DW). This pattern contrasts slightly with some earlier reports where *D*-mannoheptulose and perseitol were present in more comparable amounts [27], suggesting that environmental or genetic factors may influence the relative proportions of these heptoses. Both compounds declined sharply between the unripe and intermediate ripening stages and approached minimal levels (<1 mg/g DW) by the ready-to-eat stage. Concomitantly, glucose and fructose concentrations increased markedly, peaking at ~4–5 mg/g DW each by the ready-to-eat stage, while sucrose accumulated moderately (~3 mg/g DW). This rapid shift from C7 to C6 dominance reflects an accelerated metabolic conversion and respiratory activity in *Fuerte*, which may contribute to its sweeter flavour profile and relatively shorter postharvest life [28,29].

*Hass* fruit, although intermediate in total C7 content at harvest (~24 mg/g DW *D*-mannoheptulose, ~16 mg/g DW perseitol), displayed a distinctive dynamic compared to *Bacon* and *Fuerte*. Both C7 sugars declined consistently and were virtually absent by the ready-to-eat stage. However, unlike *Fuerte* and *Bacon*, glucose and fructose concentrations in *Hass* did not increase during ripening; rather, they showed a gradual decline from ~4 mg/g DW at harvest to ~2 mg/g DW by overripe stage. By contrast, sucrose showed moderate accumulation, rising to around ~3–4 mg/g DW at the ready-to-eat stage, after which it remained stable. This pattern suggests a tighter regulation of carbohydrate pools in *Hass*, potentially linked to its well-documented longer shelf life and controlled energy release during postharvest ripening.

The three cultivars revealed distinct metabolic strategies during ripening. *Bacon* retained C7 sugars longer than *Hass* and *Fuerte*, suggesting slower utilisation of respiratory substrates. *Fuerte* transitioned most rapidly from C7 to C6 dominance, leading to high hexose concentrations that may enhance sweetness but also potentially accelerate senescence. *Hass* maintained more balanced sugar pools, with moderate sucrose accumulation and relatively stable hexose levels, reflecting a controlled metabolic transition consistent with its widespread suitability for export and storage.

At edible ripeness, these differences were particularly marked: *Bacon* retained ~10 mg/g DW *D*-mannoheptulose, while *Hass* and *Fuerte* contained <1 mg/g DW; glucose and fructose were highest in *Fuerte* (~5 mg/g DW) and lowest in *Hass* (~2 mg/g DW). Such contrasts underscore the strong varietal component in avocado carbohydrate metabolism, independent of ripening stage.

Our findings align with several key observations from *Hass*-focused studies yet provide critical new insights by incorporating *Bacon* and *Fuerte*. Previous research has consistently reported a dominance of C7 sugars at harvest and their progressive decline during ripening [13,16,20,27,30]. The marked depletion of *D*-mannoheptulose and perseitol observed here corroborates this established pattern. However, varietal contrasts revealed in this study have not been previously documented: *Bacon*’s residual C7 sugars at ready-to-eat and overripe stages, and *Fuerte*’s rapid C7-to-C6 conversion highlight metabolic diversity beyond *Hass*.

Reports of C6 sugar dynamics in *Hass* are variable: some studies have observed increases in glucose and fructose during ripening [18], while others documented stable or declining patterns [31]). Our results for *Hass*—stable to declining hexoses and moderate sucrose accumulation—fall within this variability and may reflect differences in postharvest handling and environmental conditions. Notably, sucrose concentrations in *Hass* observed here (~3–4 mg/g DW) are intermediate between the very low levels reported by Ramos-Aguilar et al. [32] (≈0.09 mg/g DW) and the higher values (15–26 g/kg) described by Olmedo et al. [33].

By offering absolute quantification and a comparative analysis across multiple avocado cultivars under uniform ripening conditions, this study addresses a critical gap in the literature, demonstrating that sugar metabolism cannot be accurately generalised from *Hass* alone. The observed varietal differences have clear implications for breeding programmes, the development of tailored postharvest handling strategies, and the optimisation of sensory attributes and shelf life to better align with consumer expectations and market demands.

### 2.2. Dynamic Changes in Total C6 and C7 Sugar and Total Carbohydrate Content During Avocado Ripening

**Figure 1:**
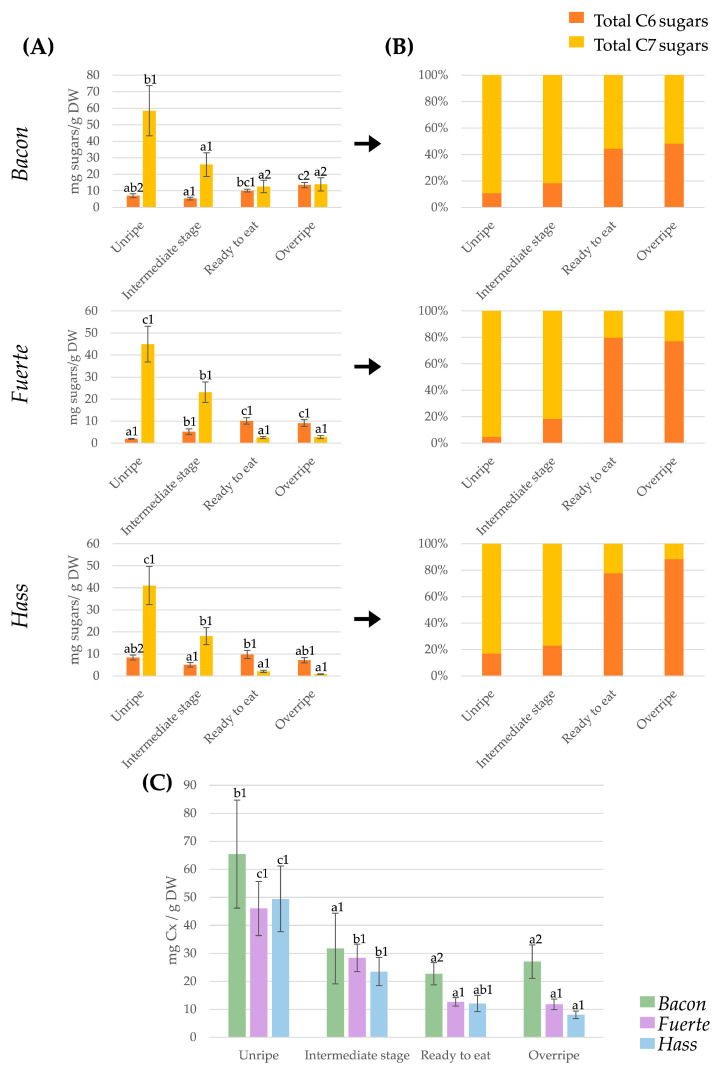
(**A**) Graph showing the evolution of C6 (sum of glucose, fructose, and sucrose) and C7 (sum of *D*-mannoheptulose and perseitol) sugars at different ripening stages for the *Hass*, *Bacon*, and *Fuerte* varieties. (**B**) Stacked bars chart representing the sum of hexoses and heptoses (as a percentage) for each ripening stage and variety. (**C**) Bar chart showing the evolution of the sum of C6 and C7 carbohydrate content at different stages of ripening in the three varieties. Different letters in bars of the same colour signify statistical disparities (*p* ≤ 0.05) between ripening stages of the same avocado variety; different numbers in bars of the same colour indicate statistically significant differences (*p* ≤ 0.05) among different varieties at the same stage of ripening.

Summation of the quantified carbohydrates by carbon number revealed clear and consistent C7-to-C6 transitions during avocado ripening (Figure 1). Figure 1 shows the total content (mg/g DW) of hexoses (glucose, fructose, and sucrose), heptoses (*D*-mannoheptulose and perseitol), and the combined C6 and C7 carbohydrates in *Bacon*, *Fuerte*, and *Hass* avocados across the different ripening stages. Specifically, Figure 1A shows the overall concentration of C6 and C7 sugars throughout the different ripening stages for each variety. Figure 1B presents this progression expressed in percentage terms, giving a more visual indication of the predominance of each type of carbohydrate at each stage, and Figure 1C presents the total sugar content for each variety at each ripening stage.

At harvest, C7 sugars dominated the soluble carbohydrate pool across all cultivars, representing > 80% of total sugars—a metabolic hallmark distinguishing avocado from most climacteric fruits, where sucrose or hexoses prevail [17,21]. Among cultivars, *Bacon* exhibited the highest total C7 content (~60 mg/g DW), followed by *Hass* (~41 mg/g DW) and *Fuerte* (~38 mg/g DW).

Throughout ripening, C7 sugar concentrations decreased substantially, with the most pronounced reductions occurring between the unripe and intermediate stages. By the ready-to-eat stage, total C7 sugar content declined to below 2.5 mg/g DW in *Hass* and *Fuerte* and to approximately 10 mg/g DW in *Bacon*, reflecting a metabolic transition from heptose- to hexose-dominated carbohydrate metabolism. This trend continued into the overripe stage, at which point C7 sugars in *Hass* and *Fuerte* approached negligible levels, while *Bacon* maintained concentrations comparable to those observed at the ready-to-eat stage. These results suggest differences in C7 sugar catabolism, mobilisation, or metabolic utilisation across cultivars.

In contrast, C6 sugars (glucose + fructose + sucrose) showed divergent patterns among cultivars. In *Fuerte* and *Bacon*, C6 levels increased progressively, rising from 4–5 mg/g DW at harvest to ~8–10 mg/g DW by the ready-to-eat stage, reflecting rapid conversion of C7 sugars to hexoses. *Hass*, however, showed stable or slightly declining C6 concentrations across ripening, indicative of higher respiratory consumption relative to C6 release.

Figure 1B shows that, in unripe fruit, C7 sugars dominate the carbohydrate pool, accounting for 83–95% of total sugars, while C6 sugars contribute only 5–17% across all cultivars. As ripening progresses, the proportion of C6 sugars rises and that of C7 sugars declines, with the most pronounced changes occurring between the intermediate and ready-to-eat stages, when hexoses surpass C7 sugars—a pattern previously reported for this transition [19]. In Bacon, hexoses reach about 48%, while in Fuerte and Hass they increase to 77% and 80%, respectively, as heptoses drop to 50%, 22%, and 10%. Importantly, this percentage shift reflects the sharp depletion of C7 sugars rather than a true rise in C6 concentrations, which remain close to those measured at harvest (Figure 1A).

Total soluble carbohydrate content (sum of C6 and C7 sugars) decreased progressively across all three cultivars from the unripe to overripe stages. This overall decline is consistent with the known role of soluble sugars—particularly C7 sugars—as primary respiratory substrates during avocado ripening. The magnitude of this reduction varied markedly among cultivars: *Hass* exhibited the most pronounced decrease (approximately six-fold), followed by *Fuerte*, while *Bacon* showed the smallest change, maintaining comparatively higher total sugar levels even in the overripe stage (in comparison with intermediate and ready-to-eat stages). While respiratory consumption is likely the predominant driver of this decline, particularly for C7 sugars, the potential contribution of other metabolic fates—such as incorporation into structural components or conversion into secondary metabolites—cannot be excluded. These cultivar-specific patterns suggest differential regulation of carbon flux during ripening.

The three cultivars exhibited distinct profiles in both absolute sugar concentrations and the trajectory of metabolic change during ripening. *Bacon* consistently maintained higher levels of C7 sugars, exhibiting a more gradual decline throughout ripening—a metabolic pattern that could be associated with extended antioxidant activity and a slower progression of ripening processes. In contrast, *Fuerte* showed rapid depletion of C7 sugars alongside a sharp increase in C6 hexoses, suggesting an accelerated ripening trajectory that may enhance sweetness but could compromise postharvest stability. *Hass* displayed intermediate initial C7 levels and relatively steady hexose concentrations, reflecting a balanced metabolic transition consistent with its well-documented postharvest resilience and suitability for global distribution.

Our findings are consistent with earlier studies on *Hass* that documented dominance of C7 sugars at harvest and their subsequent decline during ripening [16,20,27]. However, previous work rarely quantified total C6 and C7 pools across multiple cultivars, leaving unresolved whether *Hass* could serve as a proxy for avocado metabolism in general. The present study provides the first systematic evidence that varietal differences significantly influence both the rate and extent of this C7-to-C6 transition.

The total carbohydrate decline observed here parallels reports of high respiratory demand in *Hass* relative to larger-fruited cultivars like *Fuerte* [34], reinforcing the link between carbohydrate depletion and postharvest longevity. Furthermore, the persistence of C7 sugars in *Bacon* supports its reported oxidative stress tolerance [15] and highlights the potential use of C7 content as a biochemical marker for ripening and storage potential.

Under ambient storage conditions, elevated temperatures and reduced relative humidity are well-documented to accelerate carbohydrate catabolism and promote moisture loss, leading to reduced shelf life and compromised fruit quality [35]. Based on the sugar metabolism patterns observed in this study, *Fuerte* exhibited the most rapid transition from C7 to C6 sugars, consistent with a faster softening rate and potentially reduced storage capacity. *Hass* demonstrated a more moderate shift, indicative of a controlled metabolic progression, while *Bacon* maintained higher concentrations of C7 sugars for a longer period, suggesting a comparatively slower ripening response. These cultivar-specific differences highlight the importance of tailoring postharvest management strategies to each genotype in order to maintain fruit quality and extend marketability. Recommended measures include low-temperature storage (with careful avoidance of chilling injury), maintenance of high relative humidity, rapid forced-air cooling, use of moisture-retaining packaging, and, where appropriate, implementation of controlled atmosphere storage to suppress respiration and delay senescence.

By quantifying these dynamics across three major cultivars under uniform conditions, this study establishes a baseline metabolic framework for comparing avocado germplasm and developing cultivar-specific postharvest handling protocols.

### 2.3. Multivariate Analysis Highlights the Predominant Effect of Variety on the Sugar Content of Avocado Fruits

**Figure 2:**
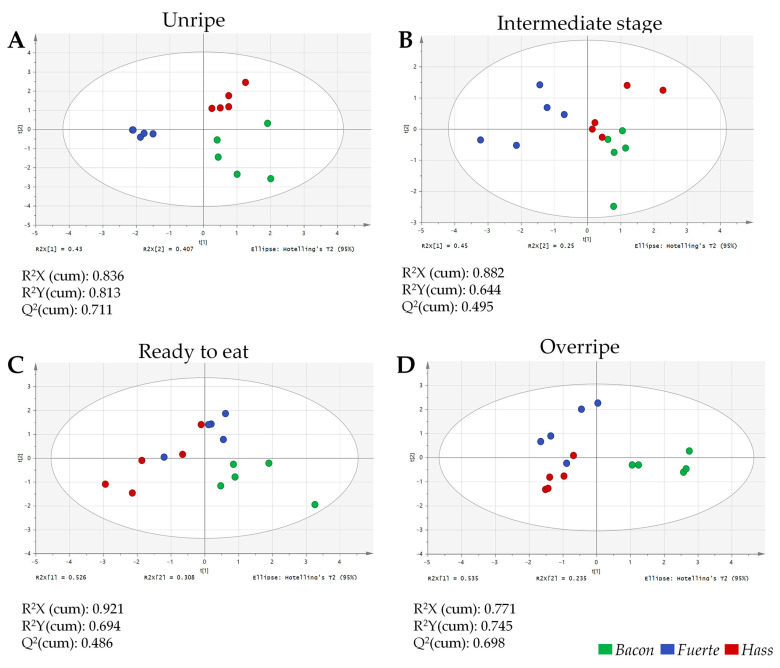
Two-dimensional PLS-DA score plots showing the differentiation of avocado varieties based on sugar profiles across four ripening stages: unripe (**A**), intermediate stage (**B**), ready-to-eat (**C**), and overripe (**D**). The R^2^X, R^2^Y, and Q^2^ values indicate the goodness of fit of each model.

PLS-DA was performed to evaluate the combined influence of ripening stage and cultivar on sugar profiles (Figure 2). As illustrated in Figure 2, the score plots of PLS-DA models constructed based on sugar profiles were employed to evaluate the capacity to differentiate between avocado varieties across four ripening stages: unripe (A), medium-ripe (B), ready-to-eat (C), and overripe (D). The plots illustrate the distribution of samples along the first two variables, with 95% confidence intervals represented by Hotelling’s T^2^ ellipses. Across all stages, variety emerged as the dominant factor separating samples.

The quality metrics of the PLS-DA models are indicated in Figure 2 for each ripening stage. In all cases, these results demonstrate that the models reliably explain the variance in both predictors (sugar profiles) and responses (variety classification), while also showing good predictive capability. The PLS-DA model for the intermediate ripening stage yielded a Q^2^ value of 0.495, while the model for the ready-to-eat stage showed a slightly lower predictive ability, with a Q^2^ of 0.486. Although these values fall near the commonly cited threshold for acceptable predictive reliability, we emphasise that these models were intended primarily for exploratory purposes—to visualise cultivar-related variation and identify discriminant metabolites—rather than for strict predictive application. Moreover, both models showed high R^2^X (0.882 for intermediate and 0.921 for ready-to-eat) and R^2^Y (0.644 and 0.694, for intermediate and ready-to-eat, respectively), indicating that the models captured a substantial proportion of the explained variance in the response variable. The lower Q^2^ values can be attributed to the biological heterogeneity of these particular stages. Intermediate ripening represents a metabolic transition phase with higher intra-group variability, while ready-to-eat fruit displays cultivar-specific physiological features that may complicate cross-sample prediction. These features reduce model predictability but are biologically meaningful. Moreover, the clustering of samples by cultivar and the consistent cross-validation results support the robustness of the model for exploratory purposes.

In the unripe stage (A), the three varieties are clearly differentiated, especially *Fuerte*, which is distinct from both *Bacon* and *Hass*. This clear distinction demonstrates that freshly harvested avocados exhibit significant disparities in their carbohydrate composition, reflecting the metabolic characteristics inherent in each variety before the ripening processes begin. At the medium-ripe stage (B), varietal separation remains detectable, though some overlap between *Bacon* and *Hass* indicates partial convergence in their sugar profiles at this stage. In contrast, *Fuerte* remains metabolically distinct from *Bacon* and *Hass* during intermediate ripening. By the ready-to-eat stage (C), *Bacon* separates clearly from the other two cultivars, while *Fuerte* and *Hass* display increasing overlap, indicating convergence of their sugar profiles. This pattern becomes even more pronounced at the overripe stage (D), where *Bacon* maintains a distinct cluster, highlighting its unique trajectory in sugar metabolism compared with the other varieties.

Overall, these findings indicate that each avocado variety follows a distinct metabolic trajectory during ripening, with varietal differences most evident at the unripe and overripe stages, whereas intermediate stages show greater convergence among cultivars. The persistent divergence of *Bacon*, particularly in the later phases, underscores that genetic background exerts a stronger influence on sugar dynamics than ripening stage alone. This highlights the value of comparing metabolic profiles across cultivars grown under identical pedoclimatic conditions, as such comparisons allow a clearer separation of genetic and environmental effects.

This insight is especially relevant in the context of the expanding avocado industry in Spain, where identifying and characterising genotypes suited to local conditions is crucial for ensuring consistent fruit quality [36]. A deeper understanding of varietal metabolic traits can guide breeding programmes and the management of genetic resources, ultimately contributing to optimised postharvest performance and improved marketability in long-distance supply chains.

## 3. Materials and Methods

### 3.1. Chemicals and Reagents

Mobile phases were prepared using doubly deionised water with a conductivity of 18.2 MΩ produced by a Milli-Q system (Millipore, Bedford, MA, USA). LC-MS grade acetonitrile (ACN) was acquired from VWR International Eurolab S.L.U. (Barcelona, Spain), and ammonium acetate was supplied by Merck Life Science S.L. (Madrid, Spain). A 0.45 μm Nylaflo^TM^ nylon membrane purchased from Pall Corporation (Ann Arbor, MI, USA) was used to filter mobile phases. Gradient grade ethanol (EtOH) from Prolabo (Paris, France) was used to prepare working solutions and for sample extraction. Standards of fructose, glucose, sucrose, perseitol, and *D*-mannoheptulose were provided by Sigma-Aldrich (St. Louis, MO, USA). Solutions containing the five target carbohydrates were prepared in EtOH:H_2_O (60:40, *v*/*v*) at twelve different concentration levels. These levels were carefully selected based on the concentration ranges observed in preliminary experiments conducted on the avocado tissues under study. In those pilot assays, all sample extracts were injected and compared against multiple concentration levels of each analyte. The results allowed us to estimate the expected range of each compound and to construct ad hoc calibration curves tailored to each metabolite. This approach ensured that all target sugars fell within the linear dynamic range of detection and allowed for accurate quantification throughout the entire dataset. Therefore, calibration curves were prepared from a mixed stock solution of all analytes (fructose, glucose, *D*-mannoheptulose, perseitol, and sucrose) in EtOH/H_2_O, 60:40 (*v*/*v*), covering the concentration ranges from the LOQ to the maximum expected concentration in avocado samples. The calibration ranges were approximately 0.29–220 mg/L for fructose, 0.22–210 mg/L for glucose, 0.34–815 mg/L for *D*-mannoheptulose, 0.10–200 mg/L for perseitol, and 0.32–100 mg/L for sucrose. External calibration curves were constructed within these ranges to ensure accurate quantification.

All the aforementioned solutions and extracts were filtered using a nylon syringe filter (0.22 μm) Clarinet^TM^ from Agela Technologies (Torrance, CA, USA) and stored in amber HPLC vials at −23 °C before analysis.

### 3.2. Samples

The fruits analysed in this study were provided by the Institute for Mediterranean and Subtropical Horticulture (IHSM-UMA-CSIC) La Mayora in Malaga (Spain), which boasts internationally unique germplasm collections of various subtropical fruit trees, including avocado. It is located at latitude 36°45′ North, longitude 4°4′ West with an altitude of 35 m above sea level. The three selected cultivars—*Bacon*, *Fuerte*, and *Hass*—are among the most widely marketed in Spain and, due to their staggered harvest periods, collectively ensure avocado availability across most of the season. *Bacon*, *Fuerte*, and *Hass* are three commercially important avocado cultivars that exhibit distinct ripening behaviours, biochemical profiles, and postharvest responses. *Hass* is the most widely cultivated variety worldwide, known for its high lipid content, slow ripening, and long shelf life. *Fuerte*, a hybrid cultivar with intermediate oil levels, tends to mature earlier and shows increased sensitivity to postharvest stress. *Bacon*, though less widely traded internationally, is valued in some markets for its smooth texture and ease of handling; it has lower lipid content. These cultivar-specific differences in physiology and composition make them well-suited for examining the genetic influence on sugar metabolism during avocado ripening.

*Bacon* fruits were harvested at the end of October 2021, *Fuerte* fruits at the end of November 2021, and *Hass* fruits in the middle of January 2022. While we acknowledge that environmental variables such as temperature, humidity, and photoperiod can vary across the harvesting period, these factors are intrinsically linked to the natural phenology of each cultivar and, as such, were considered an integral part of the varietal signature rather than a confounding variable. The aim of the study was to evaluate the cultivars under conditions that reflect their typical commercial harvest windows, thereby preserving the real-world context of their postharvest behaviour. Importantly, all fruits were sourced from the same orchards and geographical region, which minimises spatial variability and ensures consistency in preharvest agronomic practices across all samples.

The dry matter (DM) content was determined following the AOAC 920.15123 method [37] immediately after harvesting where at least 10–15 fruits were sampled to calculate the mean DM value. According to European Union (UE) regulations, avocados can be harvested when their DM content reaches a minimum threshold of 21%. Mesocarp with a DM content above 21.5% indicates that the fruit is mature and has the potential to ripen successfully, resulting in the desired characteristics. Conversely, fruit with DM values below 21.5% typically exhibits irregular ripening, undesirable characteristics, and reduced shelf-life [38]. Unripe *Bacon* fruits exhibited DM values of approximately 27 ± 2%, whereas *Fuerte* and *Hass* avocados showed DM contents of 29 ± 3% and 28 ± 2%, respectively. Following DM determination, 80 fruits per variety were selected and divided into batches of 20 to undergo controlled ripening.

Fruits at the unripe stage were processed immediately upon arrival at the laboratory, in groups of four, to generate five biological replicates per stage (n = 5, each replicate comprising four fruits). The remaining fruits were stored under well-ventilated conditions at 22 ± 2 °C with ambient relative humidity, and no exogenous ethylene exposure, for a total period of two weeks. These conditions were deliberately chosen to reflect typical ambient environments used in the commercial handling and storage of avocados, rather than highly regulated laboratory setups. Allowing minor fluctuations in temperature and humidity enhances the ecological validity of the experiment and ensures that the observed metabolic patterns are representative of real-world ripening behaviour. Intermediate-ripe fruits (firm but beginning to soften) were processed 4–5 days after harvest, ripe fruits (ready-to-eat stage) at 8–9 days, and overripe fruits (overly soft texture) at 12–14 days. To ensure comparability across cultivars with inherently different ripening kinetics, we applied consistent physiological and morphological criteria for staging. These included a combination of days after harvest, mesocarp colour, and fruit firmness. This approach ensured that samples were taken at equivalent ripening stages across cultivars.

Each biological replicate was subjected to the following preparation protocol: peeling, cutting, bagging, freezing, freeze-drying, and grinding to obtain a homogenised powder. The lyophilised avocado samples were ground using a laboratory-grade blender to produce a homogeneous powder suitable for metabolite extraction. Grinding was performed at room temperature with intermittent pulses to minimise heat buildup and preserve analyte integrity. The process continued until a fine, uniform consistency was achieved, ensuring reproducibility and consistent extraction efficiency across all samples. In total, 60 samples were produced (20 per variety, corresponding to 5 replicates × 4 ripening stages) and stored at −23 °C until analysis.

### 3.3. Carbohydrates Extraction Procedure and HILIC-MS Analyses

To extract the compounds of interest, 0.20 g of the lyophilised samples was mixed with 6 mL of EtOH:H_2_O, 60:40 (*v*/*v*) in a centrifuge tube. The tube was vortexed for 3 min and placed in an ultrasonic bath for 30 min; subsequently, it was centrifuged for 5 min at 9000 rpm, and the supernatant was separated from the solid phase. Next, the residue was subjected to a second extraction cycle using the same procedure. Finally, both supernatants were homogenised and approximately 1 mL aliquot, previously filtered, was transferred to an HPLC vial. Performing two consecutive extraction cycles ensured complete recovery of the target analytes. Everything was carried out in accordance with the previously optimised and validated extraction protocol [15].

Two complementary LC–MS platforms were employed for sample analysis. Qualitative profiling of avocado metabolites was performed using an Elute Series ultra-high-performance liquid chromatography (UHPLC) system (Bruker Daltonics, Bremen, Germany) equipped with an electrospray ionisation (ESI) source and coupled to a compact QTOF high-resolution mass spectrometer. This platform was selected for its high mass accuracy and capability to perform tandem MS (MS/MS) experiments. For quantitative analyses, an Agilent 1260 Infinity modular liquid chromatography system (Agilent Technologies, Waldbronn, Germany) coupled via an ESI source to a Bruker Esquire 2000 ion trap mass spectrometer (LC–ESI–IT MS) was used. Both instruments were fitted with a Fortis HILIC-Diol column (Fortis Technologies, Cheshire, UK), with dimensions of 2.1 × 150 mm, and 1.7 μm particle size. The injection volume was 2 μL with a flow rate of 0.3 mL/min and a working temperature of 25 °C. Mobile phases A and B were prepared with water and ACN in a ratio of H_2_O:ACN (95:5, *v*/*v*) for A and H_2_O:ACN (5:95, *v*/*v*) for B, respectively. Ammonium acetate buffer was added to achieve the same final concentration in both phases (10 mM). The gradient elution programme was as follows: 0 min, 2% A and 98% B (kept for 5 min); 20 min, 35% A and 65% B; and 21.5 min, initial conditions. Considering column reequilibration, the analysis time was close to 30 min. HILIC-MS was selected due to its high sensitivity, excellent retention of highly polar metabolites, and compatibility with non-derivatized sugars. This technique enables effective separation and quantification of structurally similar carbohydrates, including both C6 and C7 compounds, which are typically challenging to resolve using conventional reversed-phase LC methods. Coupling with mass spectrometry further enhances specificity, allowing for precise identification and quantitative profiling across complex matrices like avocado mesocarp.

ESI operated in negative polarity and Full Scan mode (within the range *m*/*z* 50–1000). The selection of negative ESI mode was not arbitrary but based on prior method development work [15] in which we systematically evaluated the ionisation efficiency of the target carbohydrates under both positive and negative polarity. Source parameters were adapted to the MS systems conditions as follows: in the IT MS system, the nebuliser gas (nitrogen) was adjusted to 30 psi, the dry gas (nitrogen) flow rate to 9 L/min, and the temperature to 300 °C. The optimum capillary voltage was set at +3200 V and the endplate offset at −500 V. In the QTOF MS system, the selected conditions were as follows: 3.0 Bar of nebuliser pressure, 9 L/min and 220 °C of drying gas and +4500 V capillary voltage. The software controlling LC-IT MS comprised Agilent ChemStation and Bruker Esquire control, whilst LC-QTOF MS used Compass Hystar and Otof Control. Data treatment was performed with Data Analysis 4.0 from Bruker Daltonics and Microsoft Excel v 2204. Given that the method had undergone full prior validation [15], a subsequent verification was performed to ensure that all quality parameters remained consistent and that the analytical performance met the stringent requirements of this study. Non-structural carbohydrate concentrations were reported as milligrams of metabolite per gram of dry weight (mg/g DW) of avocado mesocarp.

### 3.4. Statistical Analysis

The statistical analysis of the data was performed using InfoStat [39]. The nonparametric Kruskal–Wallis test was applied to compare analyte concentrations across ripening stages within each variety and to assess differences among varieties at the same ripening state. Mann–Whitney’s test was used to assess the significance (*p* < 0.05) of the differences between the mean values of the replicates. A one-way analysis of variance (ANOVA) was performed to compare the concentrations of C6 and C7 sugars in avocados of the same variety at different ripening stages and between different varieties at the same stage of ripening; the significance of the differences (*p* < 0.05) was determined using Tukey’s test.

Following initial unsupervised exploratory analyses, partial least squares discriminant analysis (PLS-DA) was subsequently performed using SIMCA v14.1 (Umetrics, Umeå, Sweden). Prior to analysis, the data matrix was standardised and scaled using unit variance. Model quality was evaluated based on goodness of fit (R^2^X and R^2^Y) and predictive ability (Q^2^) parameters. The statistical approach was chosen to resolve cultivar-specific differences in sugar metabolism across ripening stages. In the context of this study, where subtle yet meaningful shifts in C6 and C7 sugar concentrations occur over time and vary by genotype, PLS-DA was particularly well suited. This supervised method allows for dimensionality reduction and class separation, by identifying the most discriminant sugars that define each cultivar’s metabolic trajectory.

## 4. Conclusions

This study provides a detailed characterisation of C6 and C7 sugar dynamics across four ripening stages in three commercially relevant avocado cultivars—*Bacon*, *Fuerte*, and *Hass*—from orchards located in the Institute for Mediterranean and Subtropical Horticulture, managed under consistent agronomic practices, and harvested at commercially relevant times for each cultivar. By combining absolute quantification with a powerful and reliable HILIC-MS approach and multivariate analysis, we demonstrate that varietal differences dominate over ripening stage effects in shaping mesocarp sugar profiles. This finding challenges the long-standing assumption derived from *Hass*-centric studies that physiological stage alone dictates carbohydrate behaviour during ripening.

The results reveal distinct metabolic strategies: *Bacon* retains high levels of heptoses well into the ready-to-eat and overripe stages, indicating slower changes in sugar composition over ripening; *Fuerte* undergoes a rapid shift from C7 to C6 sugars, enhancing sweetness but likely shortening storage life; *Hass* displays a more balanced transition, aligning with its recognised suitability for long-distance export. These patterns provide insights into how differences in genetic background may influence carbon allocation and energy metabolism, offering a physiological context for cultivar-specific postharvest performance.

Beyond their fundamental relevance, these insights have direct practical implications. Understanding varietal sugar dynamics provides valuable tools for predicting ripening behaviour, optimising storage protocols, and refining breeding strategies aimed at balancing flavour, nutritional value, and market resilience. As avocado production expands into new regions, the ability to identify genotypes well-adapted to local pedoclimatic conditions while meeting global quality standards becomes increasingly important.

By presenting the first stage-resolved, multi-varietal sugar profiles under controlled conditions, this study establishes a robust metabolic framework for linking carbohydrate dynamics to fruit physiology and quality traits. The findings provide a scientific basis for targeted cultivar selection and the design of postharvest strategies tailored to specific metabolic characteristics, with potential benefits for consumer satisfaction and supply chain efficiency in the growing global avocado market. Moreover, this work highlights the need for future studies that integrate sugar profiling with measurements of antioxidant capacity, metabolic fluxes, and sensory properties, to validate the observed varietal differences and expand the analysis to a broader spectrum of metabolites and quality indicators.

## Figures and Tables

**Table 1:** Order of compounds, retention time, detected *m*/*z*, molecular formula, calibration curve, coefficient of determination (r^2^), and linear range for each analyte.

Compounds Order	Compound	Rt (min)	*m*/*z* Detected	Molecular Formula	Calibration Curve	r^2^	Lineal Range (mg/L)
1	Fructose	6.7	179 [M-H]^−^	C_6_H_12_O_6_	y = 1210.9x + 4767	0.9927	LOQ-220
2	Glucose	8.6	179 [M-H]^−^	C_6_H_12_O_6_	y = 1597.3x + 2349.1	0.9978	LOQ-220
3	*D*-Mannoheptulose	11.5	209 [M-H]^−^	C_7_H_14_O_7_	y = 1663.4x + 1010 y = 1179.8x + 63000	0.9976 0.9929	LOQ-51 51–815
4	Perseitol	16.8	211 [M-H]^−^	C_7_H_16_O_7_	y = 3984.1x + 7581.3	0.9933	LOQ-200
5	Sucrose	18.3	341 [M-H]^−^	C_12_H_22_O_11_	y = 1383.9x + 5001.8	0.9949	LOQ-100

Abbreviations: Rt (retention time); LOQ (limit of quantification).

**Table 2:** Content of non-structural carbohydrates (expressed as mg metabolite/g DW; mean ± SD) in mesocarp of *Bacon*, *Fuerte*, and *Hass* avocado fruits. Different letters on the same line show statistically significant differences (*p* < 0.05) between avocados of the same variety at different ripening stages; different numbers in the same column show statistically significant differences (*p* < 0.05) when comparing the same analyte at the same ripening stage in avocados of the three varieties.

***Bacon***				
	**Unripe**	**Intermediate ripening**	**Ready-to-eat**	**Overripe**
*D*-mannoheptulose	49 ± 13 ^b2^	20 ± 6 ^a2^	10 ± 4 ^a1^	13 ± 5 ^a1^
Fructose	2 ± 1 ^a,b1^	1.9 ± 0.4 ^a1^	3.6 ± 0.5 ^b,c1^	4.5 ± 0.9 ^c1^
Glucose	2 ± 1 ^a1^	2.3 ± 0.6 ^a1^	4.7 ± 0.5 ^b1^	5.3 ± 0.9 ^b2^
Perseitol	10 ± 2 ^d1^	6 ± 2 ^c1^	2 ± 1 ^b1^	0.5 ± 0.1 ^a1^
Sucrose	2.5 ± 0.9 ^a2^	1.7 ± 0.4 ^a1,2^	1.8 ± 0.4 ^a1^	3.2 ± 0.9 ^a1^
***Fuerte***				
	**Unripe**	**Intermediate ripening**	**Ready-to-eat**	**Overripe**
*D*-mannoheptulose	20 ± 6 ^c1^	8 ± 1 ^b1^	0.8 ± 0.1 ^a2^	0.8 ± 0.2 ^a2^
Fructose	0.6 ± 0.1 ^a2^	1.3 ± 0.7 ^ab1^	4.2 ± 0.9 ^c1^	3 ± 1 ^bc1^
Glucose	0.8 ± 0.1 ^a2^	1.5 ± 0.8 ^b1^	4 ± 1 ^c1^	3 ± 1 ^bc1^
Perseitol	23 ± 5 ^b2^	15 ± 4 ^b2^	1.7 ± 0.5 ^a1^	1.9 ± 0.7 ^a2^
Sucrose	0.9 ± 0.3 ^a1,2^	2 ± 1 ^b,c2^	1.6 ± 0.6 ^a,b1^	3.4 ± 0.8 ^c1^
***Hass***				
	**Unripe**	**Intermediate ripening**	**Ready-to-eat**	**Overripe**
*D*-mannoheptulose	24 ± 11 ^d1,2^	11 ± 4 ^c1,2^	1.0 ± 0.5 ^b2^	0.4 ± 0.1 ^a3^
Fructose	3.9 ± 0.7 ^b3^	2.2 ± 0.8 ^a1^	2.7 ± 0.8 ^a,b1^	1.6 ± 0.6 ^a1^
Glucose	4.0 ± 0.8 ^b1^	2.4 ± 0.8 ^a1^	3 ± 1 ^a,b1^	2.1 ± 0.7 ^a1^
Perseitol	17 ± 2 ^d2^	7 ± 2 ^c1^	1.4 ± 0.5 ^b1^	0.5 ± 0.2 ^a1^
Sucrose	0.5 ± 0.1 ^a1^	0.8 ± 0.2 ^a1^	4 ± 1 ^b2^	3.4 ± 0.9 ^b1^

## Data Availability

The raw data supporting the conclusions of this article will be made available by the authors on request.

## Supplementary Materials

The following supporting information can be downloaded at: https://www.mdpi.com/article/10.3390/plants14182856/s1, Table S1: Review of significant research focusing on various aspects such as the role, distribution, and effect on the avocado mesocarp softening of the non-structural carbohydrates (NSCs) considered in this study. These papers, presented in alphabetical order (considering first author surname), have been selected for their relevant contribution to the knowledge of avocado ripening; Figure S1: Chromatographic representation of extracted ion chromatograms (EICs) for the selected sugars: (**A**) standard mixture and (**B**) representative avocado pulp extract. Peaks: 1, fructose; 2, glucose; 3, *D*-mannoheptulose; 4, perseitol; 5, sucrose; Figure S2: Heatmap of individual sugar concentrations (mg/g fresh weight) across four ripening stages (Unripe, Intermediate, Ready-to-eat, Overripe) in three avocado cultivars (*Bacon*, *Fuerte*, *Hass*). C7 sugars (*D*-mannoheptulose, perseitol) dominate early stages and decline sharply, while C6 sugars (glucose, fructose, sucrose) display cultivar-dependent dynamics. The heatmap highlights the C7-to-C6 transition and distinct varietal signatures: *Bacon* retains higher residual C7, *Fuerte* exhibits stronger hexose accumulation, and *Hass* shows balanced profiles with late sucrose increases.
