# Supplementary material for: Cultivar-Specific Differences in C6 and C7 Sugar Metabolism During Avocado Ripening: Comparative Insights from Bacon, Fuerte, and Hass
Source: Plants (Basel). 2025 Sep 12;14(18):2856. doi: 10.3390/plants14182856 (PMC12473306; doi:10.3390/plants14182856)

Supplementary material

Cultivar-Specific Differences in C6 and C7 Sugar Metabolism During Avocado Ripening: Comparative Insights from *Bacon*, *Fuerte*, and *Hass*

María Gemma Beiro-Valenzuela[1], Romina P. Monasterio[1,2], Irene Serrano-García[1], Elena Hurtado-Fernández[3], Carmen María Sánchez-Arévalo[1], Jorge Fernando Fernández-Sánchez[1], Romina Pedreschi[4,5], Lucía Olmo-García[1], Alegría Carrasco-Pancorbo[1]*

[1] Department of Analytical Chemistry, Faculty of Sciences, University of Granada, Ave. Fuentenueva s/n, 18071 Granada, Spain; gemabv@ugr.es (M.G.B.-V.); rmonasterio@mendoza-conicet.gob.ar (R.P.M.); iserrano@ugr.es (I.S.-G.); mamens@ugr.es (C.M.S.-A.); jffernan@ugr.es (J.F.F.-S.); luciaolmo@ugr.es (L.O.-G.)

[2] Instituto de Biología Agrícola de Mendoza (IBAM), UNCuyo—CONICET, Facultad de Ciencias Agrarias, Chacras de Coria, Mendoza 5505, Argentina

[3] Department of Biological and Health Sciences, Faculty of Health Sciences, Loyola University (Universidad Loyola Andalucía), Avda. de las Universidades s/n, 41704 Dos Hermanas, Spain; emhurtado@uloyola.es

[4] Escuela de Agronomía, Facultad de Ciencias Agronómicas y de los Alimentos, Pontificia Universidad Católica de Valparaíso, Calle San Francisco S/N, La Palma, Quillota 2260000, Chile; romina.pedreschi@pucv.cl

[5] Millennium Institute Center for Genome Regulation (CRG), Santiago 8331150, Chile

* Correspondence: alegriac@ugr.es

Table S1- Supplementary material. Review of significant research focusing on various aspects such as the role, distribution, and effect on the avocado mesocarp softening of the non-structural carbohydrates (NSCs) considered in this study. These papers, presented in alphabetical order (considering first author surname), have been selected for their relevant contribution to the knowledge of avocado ripening.

Variety	Fruit ripening stage	Metabolites determined	Conclusions about the relationship between ripening and NSCs	Ref.
Pinkerton, *Hass* and *Fuerte*	Unripe fruit (May and June harvest)	*D*-mannoheptulose, sucrose, and perseitol	C7 sugars are important indicators of fruit conditions and their correlation with storability and postharvest quality. While sucrose does not significantly impact fruit ripening or postharvest quality characteristics as an energy source, it does contribute to seed maturation.	[1]
Hass	Different ripening rates: 6 – 7 days and 12 – 13 days, after harvest	Fructose, glucose, sucrose, *D*-mannoheptulose, and perseitol	Glucose and fructose levels in unripe avocados are low but increase during ripening. Sucrose concentration falls between C6 and C7, with C7 being dominant at harvest but decreasing with ripening. Slow-ripening fruit has higher *D*-mannoheptulose concentrations than fast-ripening fruit. Total C7 concentration correlates positively with fruit firmness and they act as an important energy source, carbon supplier, and antioxidant.	[2]
Hass	Unripe (freshly harvested and after cold storage) and ready-to-eat	Fructose, glucose, sucrose, D-mannoheptulose, and perseitol	After cold storage, *Hass* avocados showed significant changes in their bioactive compounds when left to ripen at room temperature. Mannoheptulose and perseitol concentrations were significantly reduced from harvest to edible ripeness, whereas sucrose levels remained constant.	[3]
Hass (from different origins)	Unripe and ready-to-eat fruit	Sucrose, *D*-mannoheptulose, and perseitol	Sucrose, *D*-mannoheptulose and perseitol were the most abundant sugars in the avocado mesocarp. The presence of *D*-mannoheptulose and perseitol played a crucial role to predict ripening stage. However, no discernible pattern was observed in the evolution of sucrose concentration in fruits from different origins. Consequently, sucrose is not a reliable indicator for assessing postharvest quality.	[4]
Hass	Unripe and ready-to-eat fruit	Fructose, glucose, sucrose, *D*-mannoheptulose, and perseitol	During fruit ripening, sugars stored in the peel or flesh are consumed, while carbohydrate reserves in the seed remain unaffected. This suggests that the energy sources stored in the seed are separate from those utilized for ripening in the flesh. Both C6 and C7 sugars decrease regardless of storage temperature or duration.	[5]
Hass	Unripe and ready-to-eat fruit	*D*-mannoheptulose and perseitol	Perseitol is the predominant soluble sugar in unripe avocado mesocarp, but its levels decrease during ripening to below those of hexoses. As result, C6 sugars represent the most abundant forms of sugar in flesh at edible ripeness. Transport of C7 sugars into the fruit may play a role in inhibiting fruit ripening while it is on the tree. The ripening process is significantly delayed until the C7 sugars in the fruit are metabolised below a specific threshold.	[6]
Hass	Unripe, medium-ripe and ready-to-eat fruit	Sucrose, *D*-mannoheptulose, and perseitol	*D*-mannoheptulose and perseitol concentrations are lower in ripe avocados compared to unripe or moderately ripe fruits, while sucrose levels show minimal variation.	[7]
Hass	5 ripening stages assessed based on firmness loss: initial softening, slight softening, ready-to-eat softness, very soft, and extremely soft	Fructose, glucose, sucrose, *D*-mannoheptulose, and perseitol	Sugar levels were linked to ripening heterogeneity. However, no significant correlations were found for C7 sugars at harvest and the time required to reach edible ripeness.	[8]
Hass	Unripe and ready-to-eat fruit	Fructose, glucose, sucrose, *D*-mannoheptulose, and perseitol	In all studied tissues of unripe avocado (mesocarp, exocarp and seed), the total amount of C6 sugars were lower than that of C7 sugars. The concentration of *D*-mannoheptulose in mesocarp decreases significantly from pre-harvest until the mesocarp softens. This suggests that *D*-mannoheptulose could serve as a useful indicator of commercial maturity.	[9]

References

1. Bertling, I.; Bower, J.P.P. Sugars as Energy Sources – Is There a Link to Avocado Fruit Quality? *South African Avocado Grow. Assoc. Yearb.* **2005**, *28*, 24–27.
2. Blakey, R.J.; Tesfay, S.Z.; Bertling, I.; Bower, J.P.P. Changes in Sugars, Total Protein, and Oil in "Hass" Avocado (Persea Americana Mill.) Fruit during Ripening. *J. Hortic. Sci. Biotechnol.* **2012**, *87*, 381–387, doi:10.1080/14620316.2012.11512880.
3. Campos, D.; Teran-Hilares, F.; Chirinos, R.; Aguilar-Galvez, A.; García-Ríos, D.; Pacheco-Avalos, A.; Pedreschi, R. Bioactive Compounds and Antioxidant Activity from Harvest to Edible Ripeness of Avocado Cv. Hass (Persea Americana) throughout the Harvest Seasons. *Int. J. Food Sci. Technol.* **2020**, *55*, 2208–2218, doi:10.1111/ijfs.14474.
4. Landahl, S.; Meyer, M.D.; Terry, L.A. Spatial and Temporal Analysis of Textural and Biochemical Changes of Imported Avocado Cv. Hass during Fruit Ripening. *J. Agric. Food Chem.* **2009**, *57*, 7039–7047, doi:10.1021/jf803669x.
5. Liu, X.; Robinson, P.W.; Madore, M.A.; Witney, G.W.; Lu Arpaia, M. "Hass" Avocado Carbohydrate Fluctuations. II. Fruit Growth and Ripening. *J. Am. Soc. Hortic. Sci.* **1999**, *124*, 676–681, doi:10.21273/jashs.124.6.676.
6. Liu, X.; Sievert, J.; Lu Arpaia, M.; Madore, M.A. Postulated Physiological Roles of the Seven-Carbon Sugars, Mannoheptulose, and Perseitol in Avocado. *J. Am. Soc. Hortic. Sci.* **2002**, *127*, 108–114, doi:10.21273/jashs.127.1.108.
7. Meyer, M.D.; Terry, L.A. Development of a Rapid Method for the Sequential Extraction and Subsequent Quantification of Fatty Acids and Sugars from Avocado Mesocarp Tissue. *J. Agric. Food Chem.* **2008**, *56*, 7439–7445, doi:10.1021/jf8011322.
8. Pedreschi, R.; Muñoz, P.; Robledo, P.; Becerra, C.; Defilippi, B.G.; van Eekelen, H.; Mumm, R.; Westra, E.; De Vos, R.C.H. Metabolomics Analysis of Postharvest Ripening Heterogeneity of "Hass" Avocadoes. *Postharvest Biol. Technol.* **2014**, *92*, 172–179, doi:10.1016/j.postharvbio.2014.01.024.
9. Tesfay, S.Z.; Bertling, I.; Bower, J.P.P.; Lovatt, C.J. The Quest for the Function of "Hass" Avocado Carbohydrates: Clues from Fruit and Seed Development as Well as Seed Germination. *Aust. J. Bot.* **2012**, *60*, 79–86, doi:10.1071/BT11166.

Figure S1-Supplementary material. Chromatographic representation of extracted ion chromatograms (EICs) for the selected sugars: **(A)** standard mixture and **(B)** representative avocado pulp extract. Peaks: 1, fructose; 2, glucose; 3, *D*-mannoheptulose; 4, perseitol; 5, sucrose.

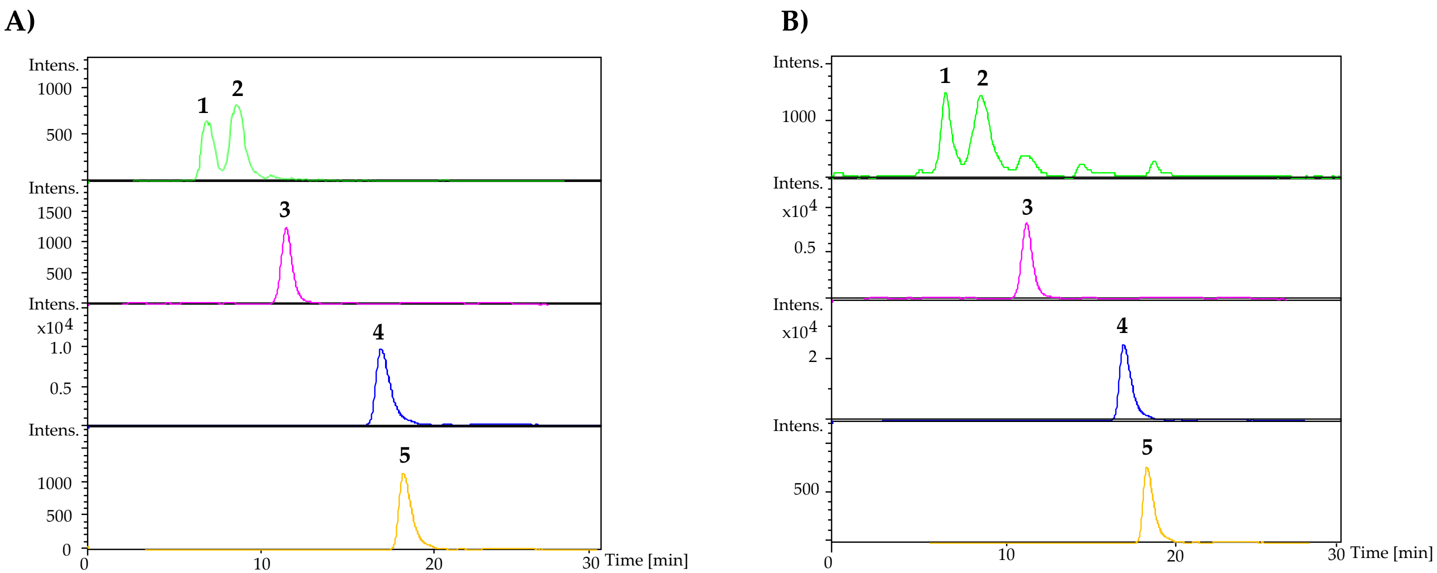

Figure S2-Supplementary material. Heatmap of individual sugar concentrations (mg/g fresh weight) across four ripening stages (Unripe, Intermediate, Ready-to-eat, Overripe) in three avocado cultivars (*Bacon*, *Fuerte*, *Hass*).

C7 sugars (*D*-mannoheptulose, perseitol) dominate early stages and decline sharply, while C6 sugars (glucose, fructose, sucrose) display cultivar-dependent dynamics. The heatmap highlights the C7-to-C6 transition and distinct varietal signatures: *Bacon* retains higher residual C7, *Fuerte* exhibits stronger hexose accumulation, and *Hass* shows balanced profiles with late sucrose increases.

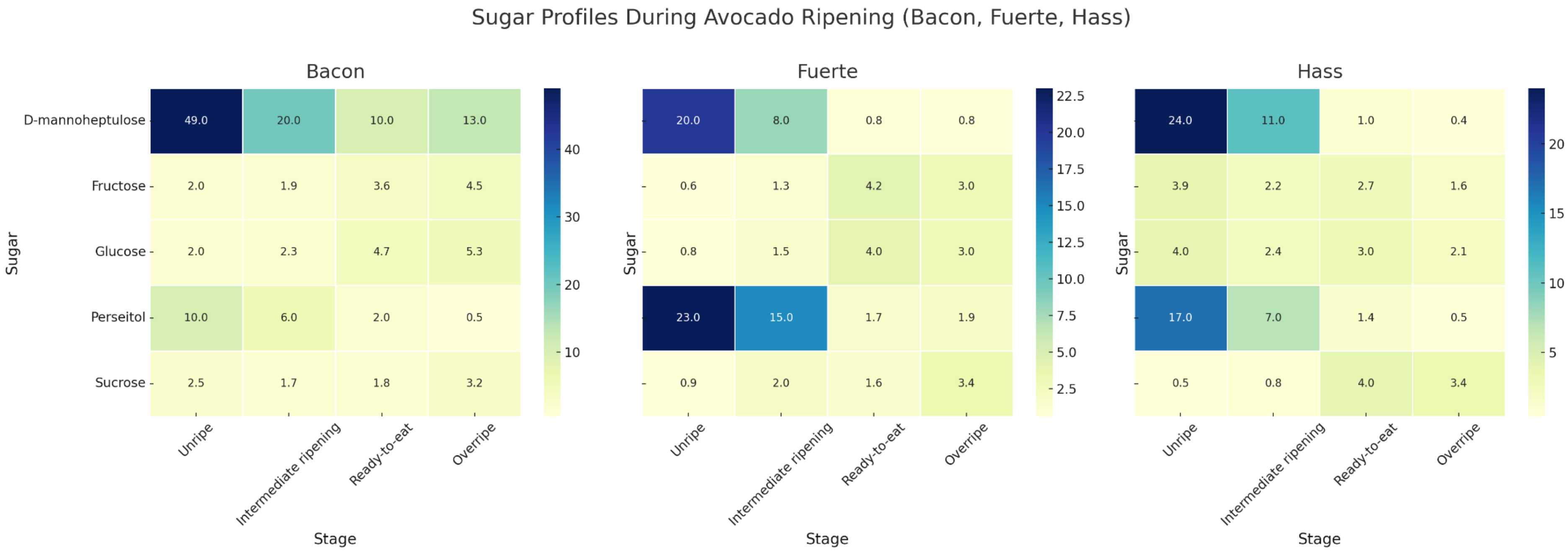

Supplement: Supplementary file 1 [file plants-14-02856-s001.zip › plants-3827443-supplementary.pdf]
